# Neurovascular Unit Dysfunction and Blood–Brain Barrier Hyperpermeability Contribute to Schizophrenia Neurobiology: A Theoretical Integration of Clinical and Experimental Evidence

**DOI:** 10.3389/fpsyt.2017.00083

**Published:** 2017-05-23

**Authors:** Souhel Najjar, Silky Pahlajani, Virginia De Sanctis, Joel N. H. Stern, Amanda Najjar, Derek Chong

**Affiliations:** ^1^Department of Neurology, Hofstra Northwell School of Medicine, New York, NY, USA; ^2^Neuroinflammation Division, Department of Neurology, Lenox Hill Hospital, New York, NY, USA; ^3^Department of Psychology and Human Development, Peabody College, Vanderbilt University, Nashville, TN, USA

**Keywords:** schizophrenia, blood–brain barrier, neurovascular unit, endothelial cell, neuroinflammation, oxidative stress, nitric oxide synthase, endothelial nitric oxide synthase

## Abstract

Schizophrenia is a psychotic disorder characterized by delusions, hallucinations, negative symptoms, as well as behavioral and cognitive dysfunction. It is a pathoetiologically heterogeneous disorder involving complex interrelated mechanisms that include oxidative stress and neuroinflammation. Neurovascular endothelial dysfunction and blood–brain barrier (BBB) hyperpermeability are established mechanisms in neurological disorders with comorbid psychiatric symptoms such as epilepsy, traumatic brain injury, and Alzheimer’s disease. Schizophrenia is frequently comorbid with medical conditions associated with peripheral vascular endothelial dysfunction, such as metabolic syndrome, cardiovascular disease, and diabetes mellitus. However, the existence and etiological relevance of neurovascular endothelial dysfunction and BBB hyperpermeability in schizophrenia are still not well recognized. Here, we review the growing clinical and experimental evidence, indicating that neurovascular endotheliopathy and BBB hyperpermeability occur in schizophrenia patients. We present a theoretical integration of human and animal data linking oxidative stress and neuroinflammation to neurovascular endotheliopathy and BBB breakdown in schizophrenia. These abnormalities may contribute to the cognitive and behavioral symptoms of schizophrenia *via* several mechanisms involving reduced cerebral perfusion and impaired homeostatic processes of cerebral microenvironment. Furthermore, BBB disruption can facilitate interactions between brain innate and peripheral adaptive immunity, thereby perpetuating harmful neuroimmune signals and toxic neuroinflammatory responses, which can also contribute to the symptoms of schizophrenia. Taken together, these findings support the “mild encephalitis” hypothesis of schizophrenia. If neurovascular abnormalities prove to be etiologically relevant to the neurobiology of schizophrenia, then targeting these abnormalities may represent a promising therapeutic strategy.

## Introduction

Schizophrenia is a pathoetiologically heterogeneous psychotic disorder characterized by delusions, hallucinations, negative symptoms, as well as behavioral and cognitive dysfunction. Current evidence suggests that schizophrenia involves complex interrelated mechanisms that influence immune, inflammatory, oxidative, neurotransmitter, and genetic pathways (1, 2). We previously reviewed the evidence implicating neuroinflammation in the neurobiology of schizophrenia, even during first-episode psychosis (1, 3). Neuroinflammation may contribute to white matter structural and functional disconnectivity, causing symptoms of schizophrenia (3). Neuropathological, biomarker, and genetic studies have documented numerous inflammatory abnormalities in individuals with schizophrenia, including microglial activation and proliferation (MAP), pro-inflammatory cytokine upregulation, and abnormal peripheral immune cell counts (1). Human PET imaging of microglial activation utilizing translocator protein (TSPO), including the second-generation TSPO radiotracer, in individuals with first-episode psychosis and recent-onset schizophrenia has yielded conflicting results. While some studies showed no alteration in TSPO ligand binding or expression (4–6), several others found it to be increased (7–9). The inconsistent findings may result from several factors. First, TSPO expression is not only selective to microglia but also includes other cells such as astrocytes and vascular endothelial cells (10). Thus, the potential negative contribution of astroglial loss or vascular endotheliopathy to TSPO expression in a subset of individuals with recent-onset schizophrenia cannot be totally excluded. Second, we suggest that central TSPO ligand binding may not be a reliable surrogate marker for low-grade neuroinflammation (10) that is typically documented in postmortem brain tissue of subjects with schizophrenia (3). Indeed, reduced TSPO binding in the middle frontal gyrus was found in individuals with recent-onset schizophrenia who were also documented to have elevated pro-inflammatory cytokines levels in both peripheral and central tissues (10). Furthermore, schizophrenia-relevant behavioral abnormalities in infection-mediated neurodevelopmental mouse model were also associated with reduced central TSPO binding despite increased pro-inflammatory cytokine levels (10). Together, these findings suggest that the lack of increased TSPO expression or ligand binding by human PET imaging in first-episode psychosis and recent-onset schizophrenia may not reliably exclude the presence of low-grade neuroinflammatory process (10).

Pro-inflammatory cytokines are thought to contribute to the pathophysiology of primary psychiatric disorders, including schizophrenia (1, 11). A meta-analysis of 40 studies including 2,572 schizophrenia patients and 4,401 controls revealed consistent elevation of serum interferon gamma (IFN-γ), TNF-α, IL-12, and sIL-2R levels in patients with chronic schizophrenia, independent of disease activity (trait markers). In addition, positive correlations were detected between elevated serum IL-6, IL-1β, and transforming growth factor beta levels and disease activity (state markers for acute psychosis) (12). Another meta-analysis found a positive correlation between increased CD4+ T-cell counts and acute psychosis in individuals with schizophrenia (13). More recently, a relative increase in naïve B-cells, natural killer cells, and monocyte counts was reported in those with schizophrenia compared to healthy controls (14). This study also showed a relative decrease in the number of CD4+ memory and human leukocyte antigen (HLA)-DR+ regulatory T-cells, which correlated with the severity of neurocognitive deficits and negative symptoms (14). Furthermore, peripheral blood mononuclear cell cultures derived from individuals with schizophrenia produced higher amounts of IL-8 and IL-1β, either spontaneously or in response to LPS stimulation, suggesting that activation of classical peripheral monocytes can contribute to the pathophysiology of schizophrenia (15).

Oxidative stress occurs in chronic and new-onset schizophrenia (1, 2, 13, 16–18). Oxidative stress markers are found in peripheral blood, neutrophils, red blood cells (RBCs), platelets, cerebrospinal fluid (CSF), and brain tissue (13, 17). Certain oxidative and antioxidative changes in schizophrenia, such as reduced catalase levels in RBCs and plasma, are state dependent and reflect disease progression, whereas others such as decreased soluble superoxide dismutase-1 levels in CSF and RBCs appear to be trait dependent (19). Human and animal studies have indicated a reciprocal cause–effect relationship between oxidative stress and neuroinflammation (1, 3).

Multiple neuropathological and neuroimaging studies have established the effects of neuroinflammation and oxidative stress on the neurovascular unit and blood–brain barrier (BBB), especially in neurologic disorders with comorbid psychiatric symptoms (16). These disorders include epilepsy, stroke, traumatic brain injury, multiple sclerosis, and Alzheimer’s disease (16). However, whether neurovascular endothelial dysfunction and BBB hyperpermeability contribute to the neurobiology of schizophrenia, causing behavioral and cognitive symptoms, remains less clear.

There is growing clinical and experimental evidence that vascular endothelial dysfunction and BBB hyperpermeability do occur in a subset of individuals with schizophrenia. However, to date, there has been no systematic attempt to synthesize and analyze the extant literature of neurovascular unit dysfunction and BBB hyperpermeability in schizophrenia. Here, we aimed to characterize the human evidence by performing a systematic review of neuropathological, neuroimaging, serological, CSF, and genetic studies relevant to this effect (Table 1).

**Table 1:** **Human and experimental data potentially linking neuroinflammation, oxidative stress, and genetic factors to clinical, laboratory, imaging, and pathological findings suggestive of neurovascular unit dysfunction and blood–brain barrier hyperpermeability in schizophrenia**.

	Summary of clinical, laboratory, imaging, and pathological findings	Mechanisms		Genetic factors
		Oxidative stress	Neuroinflammation	
Neurovascular unit dysfunction	Cerebral hypoperfusion (51, 59–62) ↓RH-PAT index <1.67 indicative of vascular endothelial dysfunction (24, 26) Epidemiological studies associating schizophrenia with peripheral vascular endothelial dysfunction (23–26) Vacuolar degeneration of neurovascular endothelial cells and astroglial end-feet processes as well as basal lamina abnormalities in postmortem PFC (31)	↓ eNOS activity-dependent oxidative endothelial effects (16, 44, 45, 53–57): – ↓ Endothelial vasodilator NO – ↑ONOO^−^ – Endothelial oxidative injury ↓ Cerebral blood flow and ↓ vascular reactivity	Astroglial cell activation and loss (1, 3, 16) ↓ AQP4 (16, 30) MAP (1, 3, 7–9, 16, 79, 82–84) Pro-inflammatory cytokines (11, 91–94) ↑ MMPs (18, 85–87)	*eNOS* T^−786^C (24) COMT Val allele (23–26) MTHFR T allele (26) ↓ Endothelial expression of genes involved in ion transport, cell proliferation, and adhesion (28)

Blood–brain barrier hyperpermeability	↑ S100B in blood, CSF, brain tissue (1, 81) CSF abnormalities (29, 32, 33): – ↑“CSF: serum albumin” – ↑ Intrathecal synthesis of IgG, IgM, IgA – ≥4 OCB – Mild pleocytosis ↑ Serum levels of vascular endothelial adhesion molecules, i.e., sP-selectin, sL-selectin, integrin αIIbβIIIa receptors on platelets (34–36) ↑ serum VEGF (40) ↓ VEGF receptor 2 expression in postmortem PFC (41) ↓ AQP4 expression in postmortem anterior cingulate gyrus (75)	eNOS-independent direct oxidative endothelial injury: – ↑ MMPs (66) – E-cadherin (44) – Altering endothelial tight junction and cytoskeleton proteins (44, 67, 68) – Inducing endothelial NR1 expression? (69) – Impairing mitochondrial oxidation (70)	Astroglial loss (1, 3, 16) ↓ AQP4 (16, 30, 75, 76) MAP (1, 3, 16, 79, 83, 84) Effects of pro-inflammatory cytokines: – Direct endothelial injury (4, 16) – Upregulating MMPs (18, 85–87) – Upregulating endothelial adhesion molecules such as ICAM-1, VCAM-1 (91–94) – Upregulating VEGF (40) – Vascular endothelial mitochondrial oxidative injury (65, 95) ↓ Ndel1 activity (96, 97) ↑ Bradykinin (29, 44, 74, 96–99) ↑ ACE activity in serum, CSF, brain (100–103)	NDST3 polymorphism—gene involved in heparan sulfate metabolism (34, 39)

*ACE, angiotensin I-converting enzyme; AQP4, aquaporin 4; CSF, cerebrospinal fluid; COMT, catechol-O-methyltransferase; eNOS, endothelial nitric oxide synthase; ICAM-1, intercellular adhesion molecule-1; Ig, immunoglobulin; MAP, microglial activation and proliferation; MMP, matrix metalloproteinase; MTHFR, methylenetetrahydrofolate reductase; Ndel1, nuclear distribution E like-1; NO, nitric oxide; NR1, NMDA receptor subunit 1; OCB, oligoclonal bands; ONOO^−^, peroxynitrite; PFC, prefrontal cortex; RH-PAT, peripheral arterial tonometry; sP, soluble P-selectin; sL, soluble L-selectin; VCAM-1, vascular cell adhesion molecule-1; VEGF, vascular endothelial growth factor*.

## Search Strategy and Methods

We performed a systematic electronic search for records indexed within MEDLINE, EMBASE, PsycINFO, or Web of Science to identify potentially eligible published peer-reviewed journal articles studies from January 2009 through February 2017. We included studies that met the following eligibility criteria: (a) neuropathological, neuroimaging, endothelium-dependent flow, cerebral perfusion or flow, serological, CSF, metabolic, and genetic studies that provided data on (b) neurovascular unit function or vascular endothelial function or BBB permeability AND (c) in individuals with schizophrenia (Table 1). We also searched for studies that met the following criteria (a) “schizophrenia AND BBB” or “schizophrenia AND neurovascular unit,” or “schizophrenia AND endothelial” AND any of the following key words: (b) neuroinflammation, microglia activation, cytokines, matrix metalloproteinases (MMPs), astroglia, inflammation, adhesion molecules, oxidative stress, reactive oxygen species (ROS), endothelial nitric oxide synthase (eNOS), cerebral perfusion or flow, bradykinin, or angiotensin I-converting enzyme (ACE) (Tables 1 and 2). We also present a theoretical integration of human and experimental data that potentially relate oxidative stress and neuroinflammation to neurovascular unit dysfunction and BBB hyperpermeability in schizophrenia (Tables 1 and 2). We discuss the relevance of peripheral inflammation to neurovascular endotheliopathy in schizophrenia patients, given the human and experimental data suggesting the potential bidirectional interaction between systemic inflammation and neuroinflammation in schizophrenia (20).

**Table 2:** **Putative mechanisms relevant to schizophrenia neurobiology that are shown in human and experimental studies to disrupt neurovascular unit function and increase blood–brain barrier permeability**.

Mechanisms	Human studies	Experimental studies
**Oxidative stress**		
eNOS uncoupling and decreased endothelial NO levels	Only indirect evidence (16, 23, 24)	(16, 43–45, 48, 50, 71–73)
ROS	(1, 2, 13, 17, 18)	(16, 44, 45, 66–69, 71)
Increased VEGF activity	(40, 41)	(42)
Cerebral hypoperfusion	(16, 51, 59–63)	(16, 53–56, 58, 64, 65)
MMP activation	(18, 85, 86)	(66, 89)
**Neuroinflammation**		
Astroglial loss and decreased AQP4	(1, 3, 75, 76)	(16, 39)
Microglial activation	(1, 3, 7–9, 79)	(16, 78, 82–84)
Pro-inflammatory cytokines	(1, 12, 94)	(4, 11, 16, 91–93)
Upregulation of adhesion molecules (ICAM-1, VCAM-1)	(34–36, 94)	(38, 91–93)
Bradykinin alteration	(96, 97)	(29, 44, 74, 98, 99)
ACE upregulation	(100, 101)	(100, 102, 103)

*ACE, angiotensin I-converting enzyme; AQP4, aquaporin 4; eNOS, endothelial nitric oxide synthase; ICAM-1, intercellular adhesion molecule-1; MMP, matrix metalloproteinase; NO, nitric oxide; ROS, reactive oxygen species; VCAM-1, vascular cell adhesion molecule-1; VEGF, vascular endothelial growth factor*.

## Neurovascular Unit Dysfunction

The neurovascular unit consists of the brain’s microvessels, pericytes, glial cells (astroglia, microglia, oligodendroglia), and neurons. It is the epicenter of several vital, tightly regulated, dynamic, and complex cellular interactions between glia, neurons, and the cerebral microvascular endothelium (16, 21, 22). Evidence indirectly linking neurovascular dysfunction to schizophrenia is derived from epidemiological data associating schizophrenia with medical conditions involving or resulting from vascular endothelial dysfunction, including cardiovascular disease, type 2 diabetes mellitus, and metabolic syndrome (23–25). About two-thirds of individuals with schizophrenia have comorbid cardiovascular disease (23, 24, 26). Smoking, poor diet, unhealthy lifestyle, chronic use of antipsychotic medication, and metabolic syndrome contribute to increased risk of cardiovascular disease and diabetes mellitus in these patients (25). Metabolic syndrome (abdominal obesity, abnormal glucose metabolism, dyslipidemia, and hypertension) accelerates atherosclerosis-related vascular endothelial dysfunction *via* metabolic, inflammatory, and oxidative pathways, independent of smoking and chronic atypical antipsychotic drug use (24, 26). In addition, some authors have documented primary peripheral vascular endothelial dysfunction in schizophrenia. The non-invasive peripheral arterial tonometry (RH-PAT)-EndoPat 2000 device has been used to assess peripheral arteriole endothelial-dependent vasodilatation (23, 24). Reduced RH-PAT values are considered clinically useful in predicting impaired peripheral arteriole endothelial-dependent vasodilatation and may reflect reduced endothelial eNOS-mediated nitric oxide (NO) synthesis (23, 24). Studies utilizing RH-PAT methodology revealed a high prevalence of vascular endothelial cell disturbance among individuals with schizophrenia (23, 24). In a prospective cohort of 83 patients with a schizophrenia spectrum diagnosis, 41 patients (50%) met the criteria for endothelial dysfunction defined as an RH-PAT index less than 1.67 (23). This effect remained statistically significant after adjusting for age, race, gender, smoking status, or atypical antipsychotic drug use (23, 24). Another study documented vascular endothelial dysfunction in medication naïve patients with schizophrenia (27). Genetic factors may also contribute to primary vascular endothelial dysfunction in schizophrenia. One study reported a strong correlation between eNOS genetic variants and endothelial functioning in individuals with schizophrenia; *eNOS* T^−786^C genotype correlated with lower RH-PAT index regardless of the presence or absence of metabolic syndrome, while CC genotype correlated with a much higher RH-PAT index only in individuals without metabolic syndrome (24). A strong association has been also demonstrated between endothelial dysfunction (RH-PAT index <1.67) and the catechol-O-methyltransferase (*COMT*) Val allele that can influence folate metabolism, regardless of other known risk factors for vascular endotheliopathy such as metabolic syndrome and chronic antipsychotic exposure (23, 24). Schizophrenic individuals carrying at least one *MTHFR* T and/or *COMT* Val risk allele have a lower RH-PAT index, reflective of greater endothelial dysfunction and lower frontal executive functions, compared with *MTHFR* CC and *COMT* Met/Met genotypes (26). These findings suggest that abnormal folate and homocysteine metabolism in association with *MTHFR* and *COMT* risk alleles can contribute to peripheral vascular and cerebrovascular endotheliopathy, thereby constituting an independent risk factor for cardiovascular disease and neurocognitive deficits in individuals with schizophrenia (26). Furthermore, lower endothelial expression of genes involved in ion transport, cell proliferation, and adhesion in schizophrenia individuals compared with healthy controls (28) lends support to the role of genetic factors in endothelial dysfunction in schizophrenia.

## BBB Hyperpermeability

The BBB consists of neurovascular endothelial cells continuously interconnected by highly functional tight junctions, pericytes, surrounding basal lamina extracellular matrix, and perivascular astroglial end-feet processes. BBB integrity is critical for maintaining brain homeostasis and immunoprotection by restricting interactions between innate and adaptive immunity (16, 21, 22, 29). Neurovascular endothelial cells play a critical role in the homeostatic regulation of cerebral microenvironment, both alone and through their complex interactions with surrounding astroglial end-feet processes and other cells (30). They regulate the efflux of toxic substances, the influx of essential nutrients, and brain ionic homeostasis. They also restrict the entry of peripheral inflammatory mediators, neuroactive substances, and water-soluble molecules into the brain (21). There is indirect evidence of BBB breakdown in schizophrenia individuals and that BBB hyperpermeability may contribute to the pathogenesis of schizophrenia (28, 29, 31, 32). This is consistent with clinical observations of increased psychosis in neurological disorders associated with BBB disruption, such as systemic lupus erythematosus, epilepsy, and autoimmune encephalitis (16).

The elevated “CSF:serum albumin ratio” in schizophrenia indicates an increased permeability of the BBB and blood–CSF barrier (29, 32, 33). A study of 63 psychiatric subjects and 4,100 controls revealed that 41% of psychiatric subjects (14 MDD and BPD and 14 schizophrenia) had CSF abnormalities, reflecting BBB hyperpermeability. These CSF abnormalities included increased intrathecal synthesis of IgG, IgM, and/or IgA, up to four IgG oligoclonal bands, and mild pleocytosis (32). Elevated S100B levels in the blood, CSF, and brains of individuals with schizophrenia are considered to be neurobiological consequences of glial activation and/or injury associated with BBB and blood–CSF hyperpermeability (1). Multiple authors have reported increased serum levels of vascular endothelial adhesion molecules such as soluble P (sP)-selectin and sL-selectin and an increased number of integrin αIIbβIIIa receptors on platelets of untreated acute schizophrenic patients compared with healthy controls (34–36). In addition, atypical antipsychotics, such as risperidone, are shown to further impair the vascular endothelial function in diabetic rats *via* activation of vascular endothelial adhesion molecules such as intercellular adhesion molecule-1 (ICAM-1), vascular cell adhesion molecule-1 (VCAM-1), and sL-selectin (37). The activation of endothelial adhesion molecules and integrins may contribute to increased transendothelial lymphocyte and monocyte migration, which in animal models correlated with cognitive and behavioral changes in response to systemic inflammation (38). Genetic factors may also influence BBB hyperpermeability and facilitate transendothelial migration of inflammatory cells in schizophrenia. A genome-wide association study has linked a *NDST3* polymorphism to an increased risk for schizophrenia (39). *NDST3* is expressed in the brain and encodes an enzyme involved in the metabolism of heparan sulfate (34). Heparan sulfate is a component of basal lamina extracellular matrix that is vital to BBB integrity. Therefore, genetically predetermined heparan sulfate abnormalities may increase BBB hyperpermeability and facilitate transendothelial leukocyte migration in some individuals with schizophrenia (34). Recent studies have documented elevated serum levels of vascular endothelial growth factor (VEGF) (40) and significantly reduced expression of VEGF receptor 2 in the prefrontal cortex (41), which likely reflects its accelerated destruction by increased levels and activity of VEGF in individuals with schizophrenia. VEGF regulates angiogenesis and increases BBB permeability (40). VEGF activation in animal models of ischemia promotes BBB disruption through endothelial endocytosis (42). These findings indicate that VEGF upregulation may contribute to BBB hyperpermeability and cerebral hypoperfusion in schizophrenia. Further support for BBB disruption comes from ultrastructural studies showing vacuolar degeneration of neurovascular endothelial cells and astroglial end-feet processes, together with thickening and irregularity of the basal lamina in the prefrontal and visual cortices of postmortem brains from schizophrenia subjects (31).

## Theoretical Integration of Oxidative and Neuroinflammatory Mechanisms

### Oxidative Stress

Reactive oxygen species minimize tissue injury and facilitate recovery at lower levels, but at high levels, they induce tissue injury by oxidizing biological macromolecules, such as DNA, proteins, and lipids (16). Common ROS include superoxide $\left({\text{O}}_{2}^{-}\right)$ and peroxynitrite (ONOO^−^). The biological effects of NO are dependent on its sources. NO produced by non-endothelial sources can be harmful and induce vascular endothelial injury through oxidative stress and inflammation (16, 43). When combined with ${\text{O}}_{2}^{-}$, NO produces highly reactive oxidant ONOO^−^, which damages the vascular endothelium and disrupts BBB integrity (44, 45). Non-endothelial NO production is mediated by neuronal NO synthase that is regulated by Ca^2+^ influx (46) and inducible NO synthase that is positively regulated by nuclear factor-kappa B signaling (47) and pro-inflammatory cytokines (48). In contrast, endothelial-derived NO is beneficial and exerts protective effects on vascular endothelial cells (16, 43). *In vitro* studies showed that endothelial-derived NO can increase cerebral blood flow by enhancing endothelium-dependent vasodilation (44, 45), inhibiting platelet aggregation by increasing endothelial cyclic guanosine monophosphate levels (44, 45), and downregulating the synthesis of vasoconstrictors such as 20-hydroxyeicosatetraenoic acid (16, 49). Endothelial-derived NO can also ameliorate vascular endothelial oxidative injury by scavenging cellular free radicals (44, 45). Endothelial eNOS mediates endothelial NO production *via* oxidative conversion of l-arginine to l-citrulline. activity of eNOS is influenced by several factors, including endothelial Ca^2+^ levels, its substrate arginine (50), and its cofactor tetrahydrobiopterin (BH_4_) (16). Reduced eNOS activity can decrease endothelial NO levels resulting in (a) reduced cerebral blood flow, (b) increased platelet aggregation, which may contribute to an increased risk of cardiovascular disease, and (c) decreased vascular reactivity due to oxidative injury of the vascular endothelium (16).

**Figure 1:**
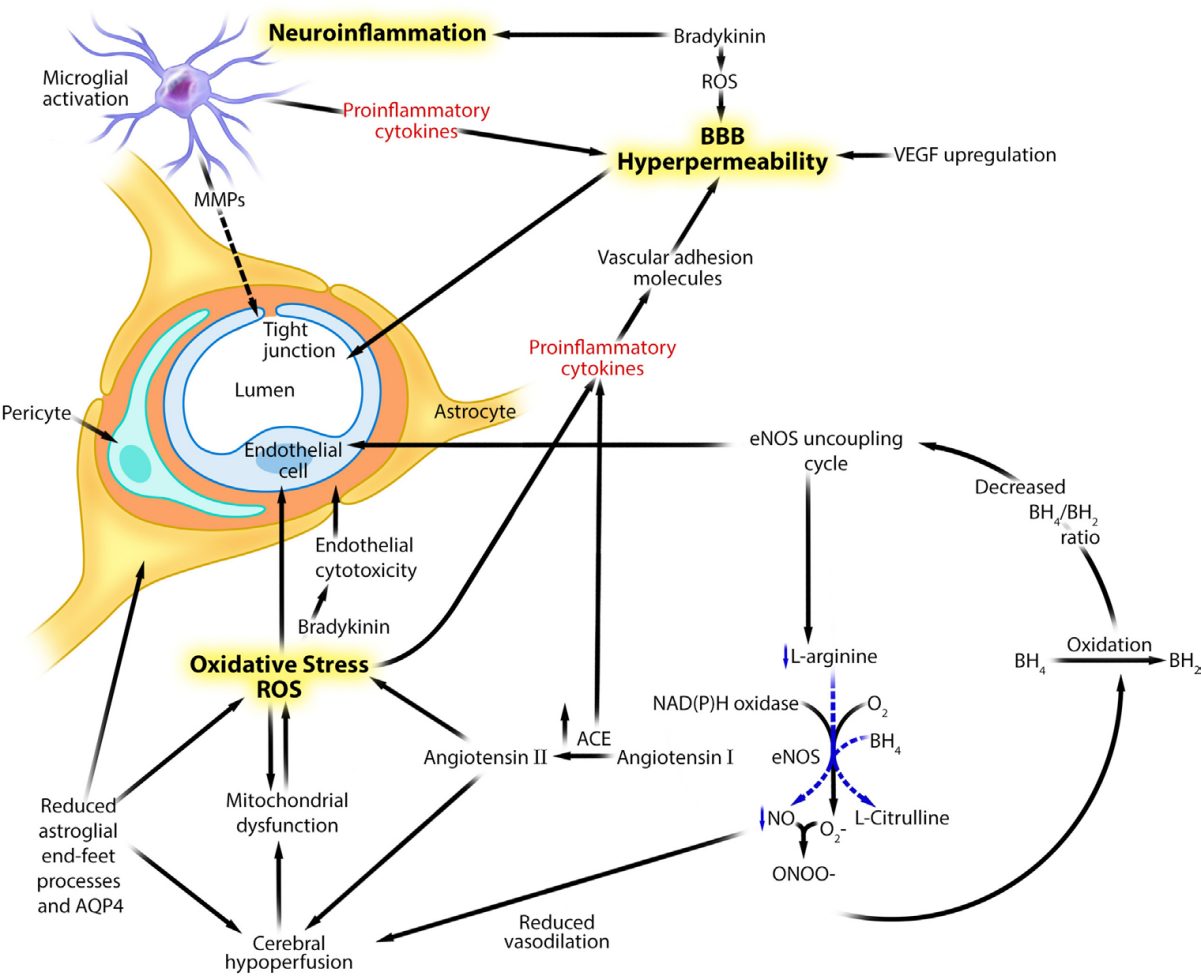
**Theoretical integration of human and experimental data linking neuroinflammation, oxidative stress, and genetic factors to neurovascular unit dysfunction and blood–brain barrier hyperpermeability in schizophrenia**. Adapted with permission from Abbott et al. (16, 21). This figure describes several putative mechanisms linking neuroinflammation, oxidative stress, and eNOS uncoupling to neurovascular dysfunction and blood–brain barrier hyperpermeability in schizophrenia. ACE, angiotensin I-converting enzyme; AQP4, aquaporin 4; BH_2_, dihydrobiopterin; BH_4_, tetrahydrobiopterin; eNOS, endothelial nitric oxide synthase; MMP, matrix metalloproteinase; NAD(P)H, nicotinamide adenosine dinucleotide phosphate; NO, nitric oxide, ONOO^−^, peroxynitrite; ${\text{O}}_{2}^{-}$, superoxide; ROS, reactive oxygen species; VEGF, vascular endothelial growth factor.

There is limited evidence for uncoupling and reduced activity of endothelial eNOS in schizophrenia (Figure 1). Reduced RH-PAT values in individuals with schizophrenia are considered indirect clinical indicators of reduced endothelial eNOS-dependent endothelial NO synthesis (23, 24). Several genetic studies have shown a significant association between eNOS gene polymorphisms and schizophrenia (24). Among 203 participants with schizophrenia or schizoaffective disorder who were carriers of the TT genotype of the *eNOS* T^−786^C variant, those without metabolic syndrome, had a lower RH-PAT index (24). A postmortem study from schizophrenia subjects showed an association between increased arginine metabolism, increased arginase II activity, and reduced eNOS expression in the frontal regions of the brain (51). We suggest that oxidation and inflammation associated with schizophrenia can also contribute to uncoupling and reduced activity of endothelial eNOS (16) (Figure 1); ROS promotes oxidative conversion of the eNOS cofactor BH_4_ to dihydrobiopterin (BH_2_), thereby reducing endothelial BH_4_ bioavailability, which in turn inhibits eNOS activity (16). Decreased BH_4_ and increased BH_2_ endothelial levels dissociate or uncouple oxidation of l-arginine from the proton-coupled electron transfer reaction, thus shifting the substrate of eNOS from l-arginine to molecular oxygen, thereby facilitating harmful ${\text{O}}_{2}^{-}$ synthesis while reducing the endothelial bioavailability of beneficial NO (16). ${\text{O}}_{2}^{-}$ combines with residual NO, to form ONOO^−^ (52), which can cause vascular endothelial oxidative injury. ONOO^−^ in turn promotes the oxidative conversion of BH_4_ to BH_2_, which further lowers eNOS activity in a positive feedback loop (52, 53).

We also suggest that uncoupling and reduced activity of endothelial eNOS may contribute to the increased risk of cardiovascular disease and neurovascular endothelial dysfunction in schizophrenia. In cardiovascular diseases, eNOS-associated endothelial dysfunction may result from (a) increased endothelial ${\text{O}}_{2}^{-}$ production through an NAD(P)H oxidase-dependent mechanism, (b) increased ONOO^−^ synthesis, (c) decreased endothelial BH_4_ bioavailability, and (d) a metabolic syndrome-related pro-inflammatory state (53–57). Furthermore, eNOS-independent direct oxidative endothelial injury can impair vasodilation (58). We suggest that similar mechanisms account for the comorbidity of cardiovascular diseases in schizophrenia. However, evidence for the potential contribution of aberrant endothelial eNOS activity to neurovascular endothelial dysfunction in schizophrenia is less direct and more limited. Regionally selective cerebral hypoperfusion abnormalities (51, 59–61), including decreased resting cerebral blood flow (62), have been documented in schizophrenia and were partly attributed to depressed neuronal activities. However, based on the findings described above, cerebral hypoperfusion may also be linked to impaired vasodilation that is mechanistically linked to reduced neurovascular eNOS-dependent NO biosynthesis (16, 51) (Figure 1). Pro-inflammatory state associated with metabolic syndrome can also impair endothelial eNOS function (24, 63). Moreover, sustained cerebral hypoperfusion can further compromise endothelial mitochondrial oxidative function, increasing the formation of endothelial ROS (64, 65), which in turn promotes eNOS uncoupling and lowers endothelial NO levels, thereby further reducing cerebral perfusion in a positive feedback loop (53–56).

In animal models, direct oxidative injury of the neurovascular endothelium has been shown to contribute to BBB disruption and cerebral hypoperfusion through several eNOS activity-independent mechanisms (Figure 1). These include (a) upregulating MMPs through upregulation of pro-inflammatory cytokines (66), (b) reducing endothelial expression of E-cadherin (44), (c) damaging BBB tight junction proteins by toxic molecules such as phosphatidylinositol-3-kinase γ (44, 67, 68), (d) altering endothelial cytoskeletal proteins, (e) inducing endothelial excitotoxicity by upregulating endothelial NMDA receptor subunit 1 expression (69), and (f) impairing endothelial mitochondrial oxidative metabolism (70). However, the mechanistic relevance of these abnormalities to the pathophysiology of schizophrenia remains unclear.

We suggest that cerebral hypoperfusion related to neurovascular endothelial dysfunction can also contribute to neuronal dysfunction and neurocognitive deficits in schizophrenia. A 99mTc-ECD-single-photon emission computed tomography brain imaging study found that schizophrenia patients with metabolic syndrome had more significant cerebral hypoperfusion associated with substantially lower frontal executive functions, compared with those without metabolic syndrome (63). This suggested a mechanistic link between neurovascular endothelial dysfunction and cognitive deficits in schizophrenia. *In vitro* animal models of selected neurological disorders have shown that reduced eNOS expression may worsen neuronal injury (71, 72). In animal models, reduced eNOS expression was associated with expansion of stroke (73), and increased levels of endothelial ONOO^−^ correlated positively with BBB breakdown and neurobehavioral deficits in traumatic brain injury (71). Furthermore, treatment with the antioxidant, *S*-nitrosoglutathione, improved neurovascular unit function by decreasing the synthesis of endothelial ONOO^−^ (71).

### Neuroinflammation

Neuroinflammation includes astroglial cell activation and loss, MAP, upregulation of inflammatory mediators, and BBB disruption with an associated increased transendothelial inflammatory cell migration. Human and animal data suggest that schizophrenia-associated neuroinflammation can disrupt neurovascular function (Tables 1 and 2; Figure 1). Astroglia regulate cerebral blood flow and volume as well as BBB permeability, among many other critical functions (16, 74). Thus, in schizophrenia, the documented loss of astroglia from functionally relevant areas, such as the subgenual cingulate, anterior, dorsolateral, and prefrontal cortices, as well as the hippocampus and corpus callosum (3), may contribute to reduced cerebral blood flow and increased BBB permeability. Aquaporin 4 (AQP4) is a bidirectional water channel mainly expressed in the perivascular astroglial end-feet processes and is critical to the development and integrity of the BBB and brain water homeostasis. AQP4 expression has been documented to be significantly reduced in the deep layers of the anterior cingulate gyrus in schizophrenia subjects (75). Decreased AQP4 expression can impair astroglial–endothelial interactions that are vital for maintaining cerebral homeostasis and regulating BBB permeability (16, 30). Furthermore, reduced AQP4 expression has been associated with an increased risk for psychiatric disorders such as psychosis (76). Thus, reduced AQP4 expression in schizophrenia individuals may contribute to neurovascular dysfunction and BBB hyperpermeability. More studies are needed to investigate the full effects of reduced AQP4 expression on the functions of neurovascular endothelium and BBB in schizophrenia.

Microglia provide immune surveillance and regulate synaptic pruning in the brain (77). Although transient MAP can limit neuronal injury and enhance recovery, persistent MAP can be harmful and perpetuate neuronal injury (78). Harmful MAP has been implicated in the pathophysiology of schizophrenia (1, 3, 79). Postmortem studies of brains from schizophrenia subjects have consistently documented MAP, including an increased HLA-DR immunoreactivity, in multiple regions compared with healthy controls, particularly in the dorsolateral prefrontal, superior temporal, and anterior cingulate cortices (3, 79). Free-water diffusion tensor imaging showed a significant increase in the extracellular free-water volume of gray and white matter in individuals with first-episode schizophrenia, suggestive of widespread neuroinflammation (80). A more recent systematic review demonstrated consistent evidence for white matter inflammation in schizophrenia individuals, which might contribute to the structural and functional white matter disconnectivity, even during first-episode psychosis (3). Furthermore, white matter inflammation has recently been associated with elevated serum S100B levels in patients with new-onset schizophrenia (81), indicating that white matter inflammation together with glial activation and/or injury as well as BBB hyperpermeability occur in early stages of schizophrenia. In experimental models of neurological diseases such as stroke and trauma, MAP damaged BBB endothelial tight junction proteins and increased BBB permeability through several mechanisms involving activation of inducible NOS (82), promotion of ROS synthesis (83), induction of COX2 expression within the neurovascular unit (1), and upregulation of pro-inflammatory cytokines and MMPs (1). An increased BBB permeability may in turn facilitate interactions between brain innate and peripheral adaptive immunity, thereby perpetuating MAP and synthesis of brain pro-inflammatory cytokines in a positive feedback loop (16). This is further supported by recent evidence linking MAP and activation of peripheral monocytes to the pathophysiology of several psychiatric disorders, including schizophrenia (84).

Matrix metalloproteinase upregulation may contribute to the pathology of schizophrenia, including neurovascular dysfunction (Figure 1). Cumulative evidence suggests that serum levels and activity of MMP-9 are increased in schizophrenia individuals compared with healthy controls (18, 85). Genetic studies have also suggested that MMP-9 may contribute to the pathology of schizophrenia (86). MMP-9 influences synaptic plasticity, thought to be relevant to the neurobiology of schizophrenia possibly by converting pro-brain-derived neurotrophic factor (BDNF) to BDNF (87). Upregulation of BDNF has been associated with resistance to antipsychotic medications (88). MMP-9 also acts *via* non-synaptic mechanisms that may be relevant to schizophrenia pathology. These mechanisms include tissue remodeling, angiogenesis, inflammation, oxidative injury, and BBB breakdown (85). In animal models of acute cerebral ischemia, upregulation of MMP-9 correlated positively with disruption and hyperpermeability of BBB (89). A positive correlation was also shown between serum levels of MMP-9 and the lipid peroxidation marker malondialdehyde in individuals with schizophrenia (18). Serum levels of malondialdehyde correlated positively with increased BBB permeability following acute neurological insults such as neonatal asphyxia (90). However, the correlation between MMP-9 upregulation, lipid peroxidation, and BBB breakdown in schizophrenia remains speculative.

Pro-inflammatory cytokines can damage and increase the permeability of the BBB (11) (Figure 1). *In vitro* data have shown that pro-inflammatory cytokines (TNF-α, IL-1β, and IFN-γ) cause a dose-dependent increase in BBB permeability by (a) inducing expression of adhesion molecules such as ICAM-1 and VCAM-1 on the luminal surface of BBB endothelial cells in animals (91–93) and humans (94), which facilitates transendothelial lymphocyte and monocyte migration; (b) causing vascular endothelial oxidative injury by impairing vascular endothelial mitochondrial oxidative metabolism (65, 95); and (c) directly damaging endothelial tight junctions (11, 16). More recently, an association was described between elevated serum IL-6 and VEGF levels in schizophrenia (40), supporting the role of inflammation in inducing BBB hyperpermeability in schizophrenia. More studies are needed to fully explore the relevance of these mechanisms to the onset and progression of schizophrenia pathology.

Bradykinin alterations in schizophrenia patients have received limited attention (96, 97). The bradykinin polypeptide mediates inflammation, prostaglandin synthesis, vasodilation, and increased capillary permeability. The oligopeptidase nuclear distribution E like-1 (Ndel1) modulates several neurodevelopmental processes involved in schizophrenia pathophysiology such as cell signaling, neurite outgrowth, neuronal migration, and cytoskeletal organization (96, 97). It also mediates the breakdown of several neuropeptides including bradykinin, which is thought to contribute to schizophrenia neurobiology (96, 97). Lower Ndel1 activity has been reported in the plasma of individuals with schizophrenia compared with healthy controls (96, 97), particularly those with treatment-resistant schizophrenia (96, 97). Therefore, lower Ndel1 activity may limit bradykinin catabolism, thereby increasing bradykinin levels in the brains of schizophrenic individuals. Upregulation of bradykinin may contribute to neurovascular endothelial dysfunction and BBB hyperpermeability *via* inflammatory and oxidative mechanisms (Figure 1). Activation of bradykinin and its inducible B1 and constitutively expressed endothelial B2 receptors induces inflammation, promotes oxidative injury, and increases BBB permeability (98). *In vitro* human studies have demonstrated that inflammation-induced expression of the bradykinin B1 receptor could increase BBB permeability (98). Bradykinin activation can augment astroglial nuclear factor-kappa B pathway-mediated IL-6 production, which may increase BBB permeability (29, 74). Bradykinin activation can also stimulate phospholipase A2 activity, which in turn enhances arachidonic acid release and metabolism, leading to increased production of malondialdehyde (99) and extracellular NO (44) that can increase BBB permeability. Endothelial B2 receptor activation increases endothelial Ca^2+^ influx, which activates pro-oxidant enzymes involved in ROS synthesis (29, 44, 74). An increased ROS production can further increase BBB permeability and augment its susceptibility to the harmful effects of bradykinin (99). *In vivo* human studies aimed at elucidating the role of bradykinin activation in schizophrenia can be informative.

Angiotensin I-converting enzyme upregulation may also contribute to neurovascular endothelial dysfunction (Figure 1). ACE is a central component of the renin–angiotensin system and converts angiotensin I to angiotensin II. Angiotensin II has vasoconstrictive and pro-inflammatory properties. ACE activity is significantly increased in the plasma, CSF, and brains of schizophrenia patients compared with healthy controls (100, 101). An increased ACE activity in schizophrenia patients correlated positively with a significant increase in the serum levels of pro-inflammatory cytokines such as IL-17 and IFN-γ (101) and cognitive deficits including disorganization of thought process (100). The pathological effects of increased ACE activity on brain, including cognitive decline, neurodegeneration and increased BBB permeability, are mediated by angiotensin II-mediated activation of angiotensin type 1 receptors (102). Activation of angiotensin II in animal models can also induce harmful cerebrovascular remodeling through inflammatory and oxidative mechanisms. These findings, collectively, suggest that ACE upregulation is relevant to the neurobiology of schizophrenia, which includes neurovascular endothelial dysfunction and increased BBB permeability (102, 103).

### Limitations and Future Directions

The evidence presented in this review suggesting a role of primary neurovascular endothelial dysfunction and BBB hyperpermeability in schizophrenia neurobiology has several limitations inherent to the following assumptions and extrapolations: (1) peripheral inflammation consistently correlates with neuroinflammation and (2) peripheral endothelial dysfunction is consistently associated with or a good surrogate marker of neurovascular endothelial dysfunction. Furthermore, although some studies suggest that neurovascular endothelial dysfunction in schizophrenia can be a primary process, many other studies support the contributory role of confounding vascular risk factors (e.g., age, BMI, smoking, metabolic syndrome, antipsychotics) to vascular endotheliopathy. Accordingly, the potential contribution of neurovascular endothelial dysfunction and increased BBB permeability to the neurobiology of schizophrenia needs to be confirmed by future investigations in animals and humans. Relevant postmortem studies should focus primarily on the neuroanatomical regions wherein astroglial loss and MAP have been consistently reported in schizophrenia, such as the subgenual cingulate, anterior, dorsolateral, and prefrontal cortices, as well as the corpus callosum (3). Future studies should concurrently investigate the potential mechanistic links between oxidative stress, neuroinflammation, aberrant expression and reduced activation of endothelial eNOS, white matter disconnectivity, and neurovascular endothelial dysfunction together with BBB hyperpermeability, in untreated new-onset schizophrenia versus chronic schizophrenia. Voxel-based morphometry could be used together with free-water diffusion tensor imaging to assess the white matter inflammation. Low RH-PAT values can be useful in predicting aberrant endothelial eNOS activity and reduced endothelial NO availability. Findings of these studies need to be correlated with serum biomarkers for inflammation (e.g., IL-6, MMP-9), oxidative stress (e.g., malondialdehyde, total antioxidant status), impaired endothelial–astroglial interaction (e.g., AQP4, S100B), and altered endothelial functions (e.g., VCAM-1, ICAM-1, sP-selectin, sL-selectin, integrin, VEGF). Furthermore, correlating *in vivo* positron emission tomography imaging of MAP using TSPO C11-PK11195 (4, 7) with serum biomarkers of vascular endothelial dysfunction and BBB breakdown may shed additional light on the role of neuroinflammation-related cerebral microvascular endotheliopathy and BBB hyperpermeability in the pathophysiology of schizophrenia.

## Conclusion

An increasing body of evidence suggests that neurovascular endotheliopathy and BBB hyperpermeability can occur in schizophrenia. Our review provides a theoretical integration of clinical and experimental findings linking neuroinflammation and oxidative stress to cerebral microvasculature abnormalities in schizophrenia. These abnormalities may contribute to the behavioral and cognitive symptoms of schizophrenia *via* several mechanisms involving disruption of BBB integrity, leading to reduced cerebral perfusion and impaired homeostatic processes of cerebral microenvironment. BBB breakdown can also facilitate interactions between brain innate and peripheral adaptive immunity, thereby perpetuating harmful neuroimmune signals and toxic neuroinflammatory responses. Taken together, these findings support the “mild encephalitis” hypothesis of schizophrenia (33). Further investigation into the molecular, functional, and structural neurovascular abnormalities and their contribution to white matter disconnectivity in untreated new-onset schizophrenia versus chronic schizophrenia, and antipsychotic treatment-responsive versus treatment-resistant schizophrenia, can be informative. If neurovascular abnormalities prove to be etiologically relevant to schizophrenia pathophysiology, then targeting these abnormalities may represent a promising therapeutic strategy for schizophrenia.

## Author Contributions

SN wrote the manuscript and was responsible for acquisition and interpretation of the data. SP, VS, JS, AN, and DC participated in the data acquisition and interpretation. All authors listed contributed to the final version of the manuscript.

## Conflict of Interest Statement

The authors declare that the research was conducted in the absence of any commercial or financial relationships that could be construed as a potential conflict of interest.
